# Evaluating the Impact of the COVID-19 Pandemic on Late-Canceled and No-Show Appointments at the Department of Neurological Surgery

**DOI:** 10.7759/cureus.60159

**Published:** 2024-05-12

**Authors:** Shawn Choe, Zachary Uram, Faraz Behzadi, Alec Germanwala, Brandon Zsigray, Omar Anwar-Hashimi, Isaac Ng, Ronak H Jani, Anand V Germanwala

**Affiliations:** 1 Neurological Surgery, Loyola University Medical Center, Maywood, USA

**Keywords:** follow-up appointment, cancellation, covid-19, no-show, general neurosurgery

## Abstract

The coronavirus disease 2019 (COVID-19) pandemic resulted in unprecedented restrictions on the general public and disturbances to the routines of hospitals worldwide. These restrictions are now being relaxed as the number of vaccinated individuals increases and as the rates of incidence and prevalence decrease; however, they left a lasting impact on healthcare systems that is still being felt today. This retrospective study evaluated the total number of canceled or missed outpatient clinic appointments in a Neurological Surgery department before and after peak COVID-19 restrictions and attempted to assess the impact of these disruptions on neurosurgical clinical attendance. We also attempted to compare our data with the data from another surgical subspecialty department. We evaluated 32,558 scheduled appointments at the Loyola University Medical Center Department of Neurological Surgery, as well as 139,435 scheduled appointments with the Department of Otolaryngology. Appointments before April 2020 were defined as pre-COVID, while appointments during or after April 2020 were defined as post-COVID. Here, we compare no-show and non-attendance rates (no-shows plus late-canceled appointments) within the respective time range. Overall, we observed that before COVID-19 restrictions were put into place, there was an 8.9% no-show rate and a 17.4% non-attendance rate for the Department of Neurological Surgery. After COVID restrictions were implemented, these increased to 10.9% and 18.3%, respectively. Greater no-show and cancellation rates (9.8% in the post-COVID era vs 8.0% in the pre-COVID era) were associated with varying socioeconomic and racial demographics. African-American patients (2.56 times higher), new-visit patients (1.67 times higher), and those with Medicaid/Medicare insurance policies (1.48 times higher) were at the highest risk of no-show in the post-COVID era compared to the pre-COVID era.

## Introduction

The first case of severe acute respiratory syndrome coronavirus 2 (SARS-CoV-2) was diagnosed in December 2019 in Wuhan, China [1]. Since then, coronavirus disease 2019 (COVID-19) has evolved into a global pandemic, disrupting daily life, economic function, and healthcare services around the world. Private practices and hospitals around the United States reported seeing fewer patients during the COVID-19 lockdown, with many hospitals limiting the number of non-emergent surgeries and procedures taking place [1-4]. This led to many healthcare systems downsizing departments or decreasing compensation for staff and providers due to the lost revenue [3]. Departments also altered the way in which they operated in an attempt to minimize both patient and staff exposure to COVID-19 [5]. Here, we evaluate the effect of the COVID-19 pandemic on outpatient clinic appointments within the neurological surgery department at a single institution and compare the results to the otolaryngology department at the same institution. We examine the rate of late-canceled or no-show outpatient clinic appointments among various patient demographics.

## Materials and methods

Patient data identification 

**Figure 1:**
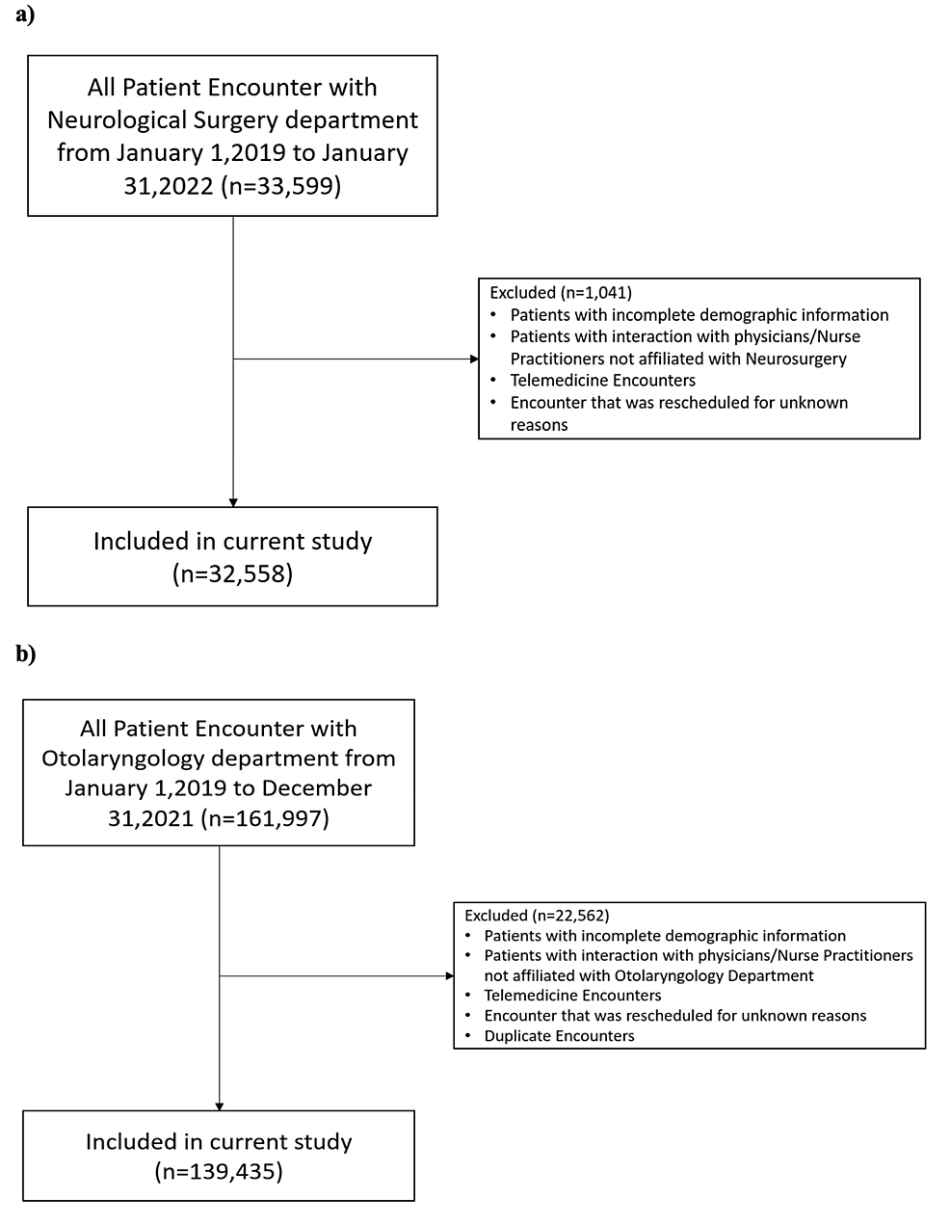
Inclusion and Exclusion Criteria for a) NSCED and b) OCED NSCED: Neurological Surgery Clinical Encounter Database; OCED: Otolaryngology Clinical Encounter Database

This study was approved under the Loyola University Medical Center (LUMC) institutional review board (IRB) with IRB number 215797. All patients who had outpatient encounters with the Neurological Surgery Department and Otolaryngology Department at Loyola University Medical Center between January 2019 and January 2022 were identified and stored in the Neurological Surgery Clinical Encounter Database (NSCED) and the Otolaryngology Clinical Encounter Database (OCED), respectively. Patients with incomplete demographic information and patients who had telemedicine encounters or rescheduled encounters were excluded from the database (Figure 1). Demographics included age, sex, appointment status, appointment type, race, ethnicity, financial class based on insurance type, preferred language, and lead time to the appointment (Table 1). We also performed a baseline comparison of neurosurgery and ENT groups to better understand possible confounders (Table 2).

**Table 1:** Demographic information of included patients in the NSCED and OCED The data within each cohort is presented as the median and interquartile range (IQR) for numerical variables and number (N) and percent (%) for categorical variables.

Variables		Neurological Surgery Clinical Encounter Database (NSCED)		Otolaryngology Clinical Encounter Database (OCED)	
		Pre-COVID (N = 14,163)	Post-COVID (N = 18,395)	Pre-COVID (N = 68,991)	Post-COVID (N = 70,444)
Age, Median (IQR)	Years	60.0 (46-71)	60.0 (44-71)	58 (37-70)	58 (38-70)
Sex, n (%)	Male	6715 (47.4)	8672 (47.1)	33179 (48.1)	32949 (46.8)
	Female	7448 (52.6)	9723 (52.9)	35812 (51.9)	37495 (53.2)
Appointment Status, n (%)	Arrived	8239 (58.2)	10903 (59.3)	44117 (63.9)	41367 (58.7)
	Canceled	4224 (29.8)	5032 (27.4)	16073 (23.3)	19202 (27.3)
	Late-Canceled	983 (6.9)	1258 (6.8)	5244 (7.6)	5567 (7.9)
	No Show	715 (5.1)	1189 (6.46)	3540 (5.13)	4244 (6.02)
	Left	2 (0.0)	13 (0.1)	17 (0.02)	64 (0.09)
Appointment Type, n (%)	New Patient Visit	3208 (22.7)	4197 (22.8)	26089 (37.8)	28833 (40.9)
	Return Patient Visit	10955 (77.3)	14198 (77.2)	42902 (62.2)	41611 (59.1)
Race, n (%)	White	9500 (67.1)	11776 (64)	50417 (73.1)	50354 (71.5)
	Black	1827 (12.9)	2440 (13.3)	6646 (9.6)	7048 (10)
	Asian	284 (2.0)	474 (2.6)	2266 (3.3)	2824 (4)
	Other/Unknown	2552 (18.0)	3705 (20.1)	9662 (14)	10218 (14.5)
Ethnicity, n (%)	Not Hispanic/Latino	12244 (68.5)	15490 (84.2)	59230 (85.9)	59922 (85.1)
	Hispanic or Latino	1919 (13.5)	2905 (15.8)	9761 (14.1)	10522 (14.9)
Financial Class, n (%)	Commercial	5254 (37.1)	7210 (39.2)	32415 (47)	32513 (46.2)
	Medicare	5862 (41.4)	7401 (40.2)	22257 (32.3)	23500 (33.4)
	Medicaid	1864 (13.2)	2760 (15)	9762 (14.1)	11014 (15.6)
	Self-Pay	353 (2.5)	368 (2)	1450 (2.1)	1516 (2.2)
	Other/Unknown	830 (5.9)	656 (3.6)	3107 (4.5)	1901 (2.7)
Preferred Language, n (%)	English	13176 (93.0)	16936 (92.1)	63762 (92.4)	64432 (91.5)
	Spanish	686 (4.8)	1082 (5.9)	2644 (3.8)	2983 (4.2)
	Other	301 (2.1)	377 (2)	2585 (3.7)	3029 (4.3)
Lead Days, Median (IQR)	Days	23 (11-49)	28 (12-57)	17 (7-45)	20 (8-44)

**Table 2:** Interdepartmental clinic attendance differences All rates and differences are provided in percent (%). NSGY: Neurosurgery

	ENT	NSGY	p-value	Mean Difference
Pre-COVID				
No-Show Rate	7.553%	8.016%	0.115217	0.46%
Non-attendance Rate	16.874%	17.158%	0.387	0.28%
Late-Cancellation Rate	23.920%	19.032%	0.000166	4.89%
Cancellation Rate	31.806%	36.479%	0.006068	4.67%
Post-COVID				
No-Show Rate	9.270%	9.820%	0.98387	0.55%
Non-attendance Rate	19.112%	18.417%	0.145017	0.70%
Late-Cancellation Rate	23.148%	20.379%	0.25505	2.77%
Cancellation Rate	34.905%	34.594%	0.447382	0.31%

Study design 

We conducted a retrospective study to compare patient encounter behavior pre-COVID defined as from January 2019 to April 1, 2020, and post-COVID defined as April 1, 2020 to January 2022. Each encounter was grouped by monthly clusters and sorted by appointment status. Analysis of the data was performed with percentile as output, and the mean difference between pre-COVID encounters and post-COVID encounters was calculated. 

$$NoShowRate = \frac{NoShowEncounters}{NoShowEncounters + CompletedEncounters}$$

$$NonAttendanceRate=\frac{NoShowEncounters+LateCancellationEncounters}{NoShowEncounters+LateCancellationEncounters+CompletedEncounters}$$

$$LateCancellationRate=\frac{LateCancellationEncounters}{LateCancellationEncounters+CancelledEncounters}$$

$$CancellationRate=\frac{LateCancellationEncounters CancelledEncounters}{LateCancellationEncounters+CancelledEncounters+NoShowEncounters+CompletedEncounters}$$

Statistical analyses 

Variables were described categorically based on the encounter data. No-shows were defined as missed appointments with no communication. Late cancellations were defined as appointment cancellations within 24 hours of the scheduled appointment. Non-attendance was defined as the sum of no-shows plus late-canceled appointments, defining all patients who did not attend their expected clinic visit with limited notification to the provider. No-show rates, non-attendance rates, late cancellation rates, and cancellation rates in pre- and post-COVID groups were compared and analyzed for mean values using independent Student’s t-tests. Line graph plots were constructed to visualize each of the rates calculated from categorical data derived from appointment status. Multivariate logistic regression was analyzed using Wald tests. Multivariate logistic regression analysis on both databases was performed based on the calculated criteria for no-show rates, non-attendance rates, late cancellation rates, and cancellation rates. Regression model variables were age, sex, appointment status, appointment type, race, ethnicity, financial class based on insurance type, preferred language, and lead time to the appointment. A forest plot was created based on multivariate logistic regression analysis and labeled based on the Wald test for logistic regression by significance. The area under the curve (AUC) for each model was quantified and compared between pre-COVID and post-COVID encounters for each calculated rate. Statistical significance was defined as a p-value < 0.05. All statistical analyses were performed using R Statistics version 4.1.2 (R Core Team 2021; R Foundation for Statistical Computing, Vienna, Austria). 

## Results

**Figure 2:**
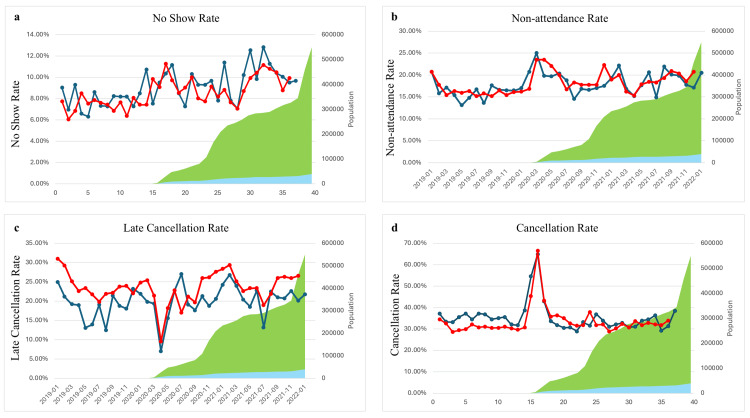
(a) No-show rate, (b) non-attendance rate, (c) late cancellation rate, and (d) cancellation rate calculated over time (red and blue represent the OCED and NSCED respectively; green represents cumulative COVID-19 Cases in Chicago; yellow represents cumulative COVID-19 hospitalizations in Chicago)

A total of 32,558 encounter records with 9,959 unique patients were included in the NSCED and 139,435 encounter records with 37,693 unique patients were included in the OCED. The no-show rate difference was very similar between the two departments during both pre-COVID and post-COVID time periods (mean difference 1.8% for the NSCED p = <0.001 vs 1.7% in the OCED p = <0.001) (Table 3). Patients in the OCED showed a statistically significant increase from pre-COVID to post-COVID non-attendance rate (2.24%, p =0.004), while patients in the NSCED showed only a slight increase that was not statistically significant (1.25%, p = 0.150). New patients had no statistically significant difference in no-show and non-attendance rates between pre- and post-COVID eras for both the NSCED and the OCED. However, return patients showed a statistically significant increase of 2.07% (p < 0.001) in no-show rates post-COVID (Table 4 and Table 5). Patient sex and age had no impact on the non-attendance rate. The rates of cancellation are represented in Figure 2.

**Table 3:** Average and mean difference of calculated rates between the NSCED and OCED The data within each cohort is presented as a percentage of total and percent difference. Statistical significance cut-off of p<0.05. NSCED: Neurological Surgery Clinical Encounter Database; OCED: Otolaryngology Clinical Encounter Database

Variables	Neurological Surgery Clinical Encounter Database				Otolaryngology Clinical Encounter Database			
	Pre-COVID	Post-COVID	Difference (%)	p-Value	Pre-COVID	Post-COVID	Difference (%)	p-Value
No-Show Rate, %								
	8.013	9.816	1.8	<0.001	7.553	9.27	1.7	<0.001
Non-attendance Rate, %								
	17.15	18.41	1.25	0.15	16.87	19.11	2.24	0.004
Late-Cancellation Rate, %								
	19.03	20.38	1.35	0.331	23.92	23.15	-0.77	0.574
Cancellation Rate, %								
	36.47	34.59	-1.89	0.409	31.81	34.91	3.1	0.172

**Table 4:** Multivariate logistic analysis of the no-show rate in the NSCED The data within each cohort are presented as the odds ratio with an appropriate confidence interval (CI). Statistical significance cut-off of p<0.05. NSCED: Neurological Surgery Clinical Encounter Database

No-Show Rate		Pre-COVID				Post-COVID			
		Adjusted Odds Ratio	CI (95%)		p-Value	Adjusted Odds Ratio	CI (95%)		p-Value
			Lower	Upper			Lower	Upper	
Sex	Male vs Female	1.20	1.01	1.43	0.036	1.16	1.01	1.33	0.035
Patient Type	Return vs New	1.560	1.240	1.982	<0.001	1.669	1.377	2.021	<0.001
Race	African American or Black vs Caucasian or White	2.56	2.05	3.19	<0.001	2.22	1.85	2.66	<0.001
	Asian vs Caucasian or White	1.09	0.52	2.03	0.799	0.98	0.56	1.60	0.940
	Others vs Caucasian or White	2.52	1.75	3.55	<0.001	1.85	1.35	2.50	<0.001
Ethnicity	Non-Hispanic vs Hispanic	1.59	0.97	2.19	0.068	1.13	0.80	1.59	0.474
Financial Class	Medicare/Medicaid vs Commercial	1.40	1.15	1.61	<0.001	1.48	1.28	1.73	<0.001
	Self-Pay vs Commercial	5.50	3.57	8.37	<0.001	3.90	2.68	5.64	<0.001
Preferred Language	Spanish vs Eng	0.73	0.46	1.11	0.150	1.29	0.96	1.73	0.088
	Others vs Eng	1.58	0.90	2.64	0.093	1.22	0.71	1.97	0.448

**Table 5:** Multivariate logistic analysis of the no-show rate in the OCED The data within each cohort are presented as the odds ratio with an appropriate confidence interval (CI). Statistical significance cut-off of p<0.05. OCED: Otolaryngology Clinical Encounter Database

No-Show Rate		Pre-COVID				Post-COVID			
		Adjusted Odds Ratio	CI (95%)		p-Value	Adjusted Odds Ratio	CI (95%)		p-Value
			Lower	Upper			Lower	Upper	
Sex	Male vs Female	0.99	0.91	1.07	0.81	1.06	0.99	1.14	0.097
Patient Type	Return vs New	1.14	1.05	1.25	0.003	0.88	0.81	0.95	0.001
Race	African American or Black vs Caucasian or White	3.10	2.78	3.45	<0.001	2.57	2.32	2.85	<0.001
	Asian vs Caucasian or White	1.07	0.84	1.34	0.57	1.11	0.91	1.35	0.299
	Others vs Caucasian or White	1.37	1.21	1.55	<0.001	1.37	1.23	1.52	<0.001
Ethnicity	Non-Hispanic vs Hispanic	0.62	0.54	0.70	<0.001	0.71	0.63	0.79	<0.001
Financial Class	Medicare/Medicaid vs Commercial	1.21	1.12	1.32	<0.001	1.44	1.34	1.56	<0.001
	Self-Pay vs Commercial	4.30	3.46	5.30	<0.001	3.23	2.63	3.94	<0.001
Preferred Language	Spanish vs Eng	1.00	0.83	1.20	0.99	1.16	0.98	1.37	0.086
	Others vs Eng	1.35	1.10	1.64	0.00	1.36	1.14	1.60	<0.001

To further characterize the demographics of patients who missed appointments, we performed a multivariate logistic regression. Odds ratios were calculated for multiple demographics. Within the multivariate logistic regression analysis in the NSCED, returning patients, African American patients, patients on Medicare/Medicaid or with no insurance, and non-English speaking patients were statistically more likely to miss their appointments (no-show) in both the pre- and post-COVID eras (Table 3). Return patients were 1.669 times as likely to no-show than new patients (odds ratio 95% CI 1.377 - 2.021, p < 0.001). African American patients were 2.22 times more likely than Caucasian patients to no-show (95% CI 1.85 - 2.021, p < 0.001). Within the OCED, African American patients, patients on Medicare/Medicaid or with no insurance, and non-English speaking patients were also associated with a higher no-show rate across both time periods, but the rates did not change significantly due to COVID (Table 5). 

**Figure 3:**
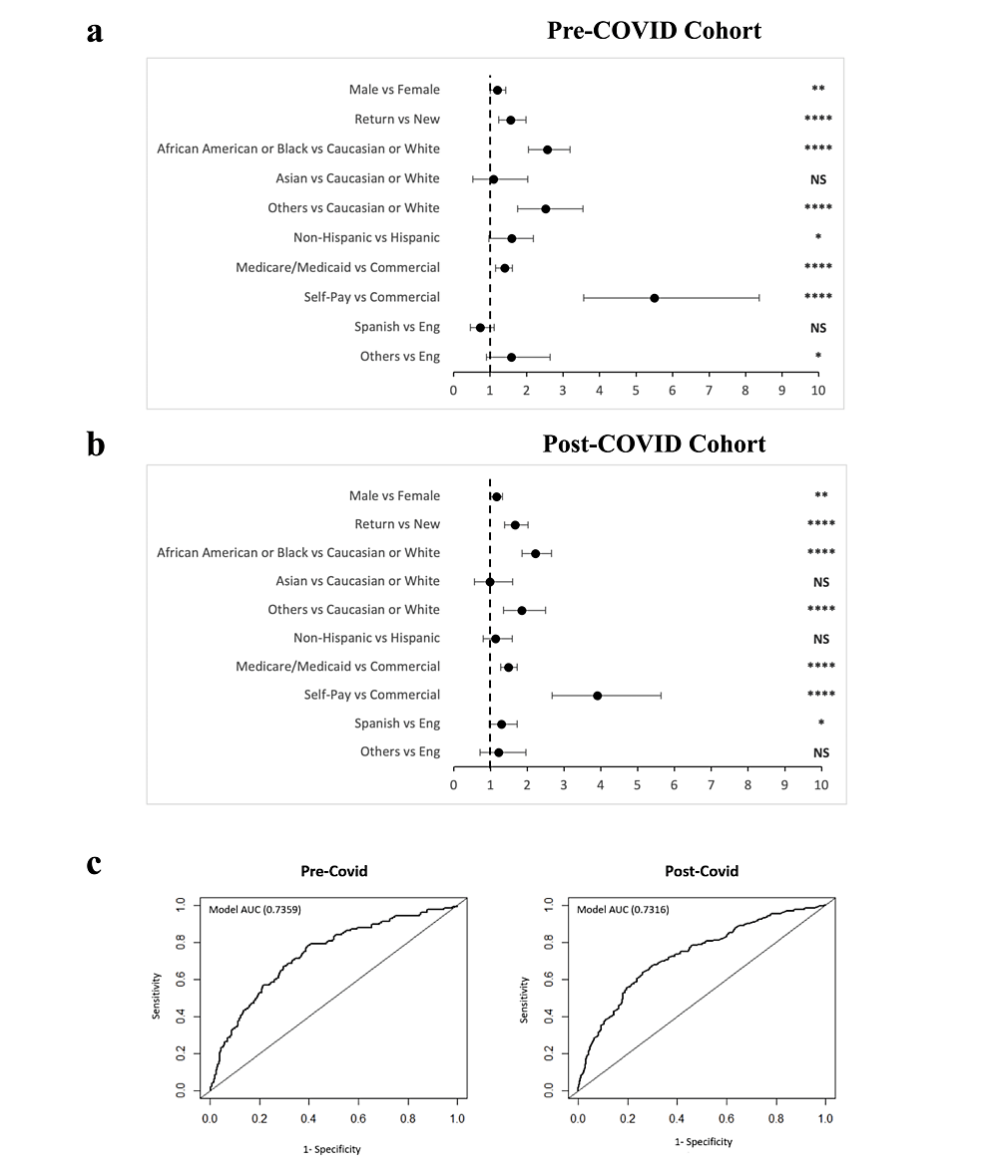
Multivariate logistic regression analysis of no-show rates in the NSCED. Forest plot of the adjusted odds ratio based on no-show rates in a) pre-COVID cohort and b) post-COVID cohort (Wald Test, p< 0.1 *, p<0.05 **, p <0.01 ***, p <0.0001 ****), and c) receiver operating curve for the multivariate logistic regression analysis model. AUC (area under the curve) Chi-square of the model compared with chance p<0.0001 NSCED: Neurological Surgery Clinical Encounter Database

**Figure 4:**
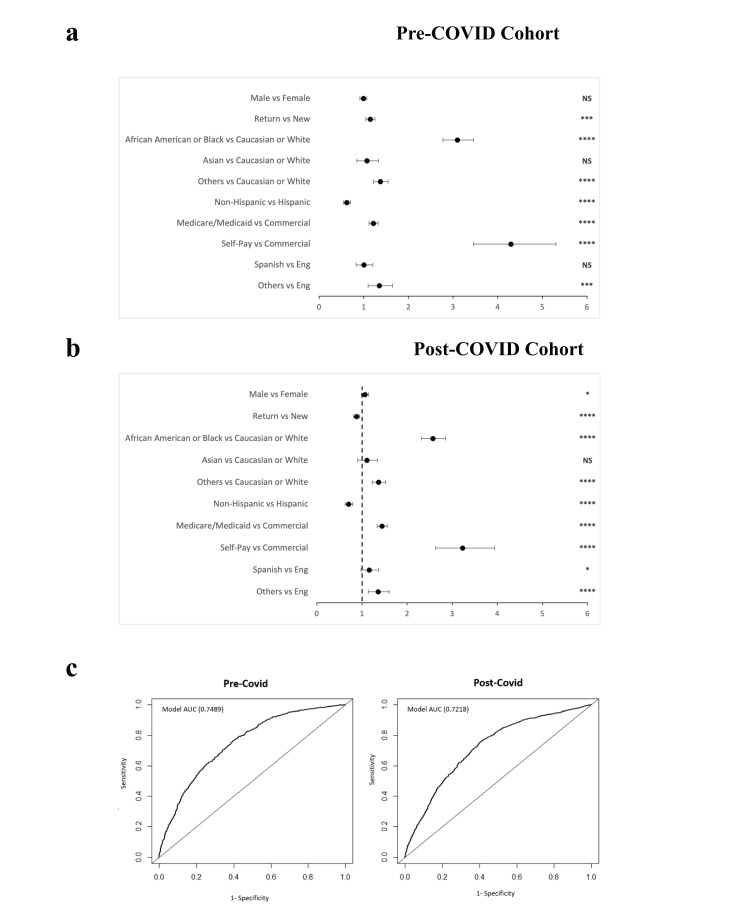
Multivariate logistic regression analysis of no-show rates in the OCED. Forest plot of the adjusted odds ratio based on no-show rates in a) pre-COVID cohort and b) post-COVID cohort (Wald Test, p< 0.1 *, p<0.05 **, p <0.01 ***, p <0.0001 ****), and c) receiver operating curve for the multivariate logistic regression analysis model. AUC (area under the curve) Chi-square of the model compared with chance p<0.0001 OCED: Otolaryngology Clinical Encounter Database

Similar results for the multivariate logistic regression analysis were shown for the non-attendance rate when compared to no-show rates. Self-paying patients in both pre-COVID and post-COVID eras were 2.95 times more likely compared to commercial payors to cancel their appointment within 24 hours of their scheduled appointment (95% CI 2.85 - 5.24, p < 0.001). African American patients in the post-COVID NCSED population were 1.62 times more likely to have a late cancellation (95% CI 1.41 - 1.86, p < 0.001); African American patients in the post-COVID OCED population were 2.03 times more likely to have a late cancellation (95% CI 1.89 - 2.19, p < 0.001). Overall, self-paying patients were the most likely to cancel. Multivariate logistic regression adjusted odds ratios were visualized in forest plots and were validated with AUC curves (Figures 3, 4). 

## Discussion

While much of our data indicates that there was little-to-no difference in attendance rates between pre- and post-COVID populations, there were a few consistent patterns in patient populations. We determined that certain demographics were more likely to miss their appointments without first canceling them. First, African American patients in the NSCED were more likely, both pre- and post-COVID, to have a no-show appointment [6-8]. We also found that patients who were on Medicare/Medicaid or were paying for procedures out of pocket were much more likely to have a no-show appointment than patients with commercial insurance. These patterns were relatively consistent for both the Department of Neurological Surgery and the Department of Otolaryngology. 

Although there were few differences in attendance rates between the two departments, we believe that the larger patterns are useful to recognize. According to multiple studies, consistent care and higher patient activation lead to decreased healthcare resource utilization, increased patient satisfaction, and overall improved outcomes [9,10]. Our results identify populations of patients who are at a higher risk of having gaps in their health care, which inevitably leads to worse outcomes, decreased patient satisfaction, and lower quality of life. By identifying these at-risk patient groups early, departments can improve their patient outreach, and perhaps their general approach to appointment scheduling, to prevent these undesirable consequences. Possible solutions include text/call reminders to patients, ensuring that patients understand their follow-up instructions, and increasing the availability of Telehealth visits; each institution may have varying capabilities for implementing such strategies [11]. Within our institution, for example, patients have access to a user-friendly electronic messaging interface called "My Loyola" where they can directly text their providers with their questions and concerns; this platform has saved patients from long emergency department wait times and stressful clinical scenarios. Ultimately, improved appointment attendance rates not only lead to improved outcomes but also contribute to a more efficient practice. Telehealth is also a relatively new phenomenon in the post-COVID era that has improved the efficiency of care for many patients. However, telehealth has limited use only in return visit patients and has many clinical barriers to a comprehensive quality of care. We believe that although telehealth may have influenced our results here, it has not replaced the key role of an in-person clinic visit with the patient.

Our data indicate that COVID had a minimal impact, if any, on the Department of Neurological Surgery’s outpatient clinic appointment attendance rate. While our non-attendance rate did appear to increase, this increase was not statistically significant. The relatively small increase in missed appointments for the Department of Neurological Surgery may in part be due to the nature of surgical specialties. Surgical issues generally must be evaluated in person, with an in-depth, complete physical exam almost always supplemented by imaging. Although telehealth visits can be beneficial for non-procedural patient interactions, and our department did increase the number of telehealth visits during the post-COVID era, surgical specialties in general cannot provide definitive surgical care via telehealth, which may be protective even against global pandemic-induced census decreases [12].

Limitations 

Given the large increase in the number of telehealth visits during the COVID-19 pandemic, excluding patients who rescheduled their visits to a telehealth visit might seem to represent a large exclusion. However, these are patients who were ultimately seen by a provider and thus should not have a no-show or late-canceled appointment. 

Future directions 

Evaluating patient-reported reasoning for missing appointments may provide some insight into common difficulties patients face in making their appointments. Sending a survey to patients who recently had a late-canceled or no-show appointment might be an effective method of probing into the cause of these missed appointments. Gaining deeper understanding could allow departments to implement strategies to assist patients in making their appointments. Specifically, a recent study found that targeted text message reminders were able to significantly decrease the no-show rate among patients in primary care specialties [13].

It may also be beneficial to evaluate the financial impact these missed appointments had on the Department of Neurosurgery and the Department of Otolaryngology during the post-COVID era. Future research will be necessary to quantify the financial losses sustained and to correlate those losses with departmental downsizing and changes in department dynamics. Each payor class is associated with a different average cost per neurosurgical procedure, so the total loss of revenue of each department is difficult to estimate. 

Finally, evaluating the rate of telehealth visits among patients with canceled appointments would provide greater insight into the increased cancellation and no-show rates observed across specialties. It is possible that the increased rate of non-attendance matches with the increase in telehealth visits; further research would be required to evaluate this possibility. Most importantly, in a new telehealth landscape of care, there may be less need for physical clinic spaces and nursing staff, reducing the initial hospital costs; however, a less comprehensive visit without a thorough physical exam may cost the hospital more in readmissions and other complications. Therefore, it is important that future studies tackle these subjects by studying the hospital costs (from emergency department visits, readmissions, and complications) of missed appointments in these eras.

## Conclusions

The COVID-19 pandemic had a minimal impact on no-show and late-canceled appointments at the Loyola University Medical Center Department of Neurological Surgery and Department of Otolaryngology, as evidenced by our data. Our data demonstrates that certain populations of patients, including African American patients, non-English-speaking patients, and patients on Medicare/Medicaid or without insurance were more likely to miss appointments. We propose that departments should develop their patient outreach strategies to ensure that these patients are connected to resources that may optimize appointment attendance rates. Doing so would facilitate benefits not only for the patients and their health but also the departments and the physicians themselves.
